# Newly graduated registered nurses' self‐assessed clinical competence and their need for further training

**DOI:** 10.1002/nop2.443

**Published:** 2020-01-22

**Authors:** Anna Willman, Kaisa Bjuresäter, Jan Nilsson

**Affiliations:** ^1^ Department of Health Sciences Faculty of Health, Science, and Technology Karlstad University Karlstad Sweden; ^2^ Department of Health Promotion Sciences Sophiahemmet University Stockholm Sweden; ^3^ Japanese Red Cross Institute for humanitarian Studies Tokyo Japan

**Keywords:** acute care settings, clinical competence, competence development, complex patient situations, newly registered graduated nurses, nurse competence

## Abstract

**Aim:**

To explore and describe changes in self‐assessed clinical competence and the need for further training among newly graduated Registered Nurses during their first 15 months of professional work in acute care hospital settings.

**Design:**

Quantitative longitudinal design.

**Methods:**

The 50‐item Professional Nurse Self‐Assessment Scale of clinical core competencies II was used. A total of 45 newly graduated Registered Nurses answered the questionnaire at four different occasions. Data were collected after 2, 5, 9 and 15 months of working experience.

**Result:**

The components “ethical decision‐making,” “cooperation and consultation” and “clinical leadership” were rated highest in clinical competence and lowest in need for further training. The components “professional development” and “critical thinking” were rated lowest in clinical competence and “direct clinical practice” rated highest in need for further training. The clinical competence increased significant between 9–15 months, with the exception of “critical thinking” and need for further training decreased significantly between 9–15 months, with the exception of “critical thinking.”

## 1 INTRODUCTION

Globally, health care has gone through significant changes in recent decades and is still rapidly changing (Butterworth, 2014). Furthermore, health care is becoming increasingly specialized and complex (Disch et al., 2016; Musau, Baumann, Kolotylo, O'shea, & Bialachowski, 2015). Concurrently, healthcare systems all over the world are being challenged by a shortage of nurses (ICN, 2017) and especially of experienced nurses (National Board of Health & Welfare, 2015). These conditions place new demands on the competence of newly graduated Registered Nurses (NGRNs), as nurses' competence is crucial to patient safety (Aiken, Sermeus et al., 2012; Aiken, Sloane et al.,^2014^, ICN, 2019). The goal of health care is to give safe and high‐quality care and place nurses' competence in focus (ICN 2012, 2019). The clinical context where nursing care is carried out is changing rapidly (Lima, Newall, Kinney, Jordan, & Hamilton, 2014). For instance, the number of patients admitted to hospitals with comorbidities and multi‐system disorders has increased, which is both challenging and it places new demands on NGRNs (Sturmberg & Lanham, 2014). Kentischer, Kleinknecht‐Dolf, Spirig, Frei, and Huber (2018) highlighted that further research into NGRNs' competence in an increasingly complex healthcare system such as acute care hospital settings is needed.

## 2 BACKGROUND

### 2.1 Transformation of health care

Several Western countries (Buchan, O'may, & Dussault, 2013) including Sweden (National Board of Health & Welfare, 2015) are going through a transformation in their healthcare system. This transformation is multifaceted due to factors such as decreased patient length of stay (Buchan et al., 2013) and patient morbidity becoming more complex due to an increase in both chronic and acute diseases (Dharmarajan et al., 2016). Patients who have previously been treated in intensive critical care departments often receive care in general hospital settings such as medicine and surgery wards nowadays (Massey, Aitken, & Chaboyer, 2009). Hospital‐acquired infections have become increasingly common (Musau et al., 2015), and new medical interventions and technology are evolving at higher rates than ever (Buchan et al., 2013). The complexity of nursing care is dynamic and can be understood as the interaction of diverse components relating to personal, social and clinical features (Shippee, Shah, May, Mair, & Montori, 2012) that make the nursing situation more unpredictable and therefore more complex (Reed, 2013).

The shortage of nurses is a global concern (ICN, 2019; WHO, 2018), caused by factors such as extensive generation change of staff (National Board of Health & Welfare, 2015), job disaffection, stress and high workloads, which in turn further increases turnover and reduces the number of nurses (Aiken et al., 2012; Laschinger et al., 2016; Rudman & Gustavsson, 2011; Rudman, Gustavsson, & Hultell, 2014). This shortage of nurses and especially of experienced nurses has a negative impact on quality of care and patient safety and is associated with a negative impact on nurses' competence and job satisfaction. As a result of the lack of experienced nurses, a greater proportion of inexperienced NGRNs is in the workforce and their formal education alone does not give full professional competence (National Board of Health & Welfare, 2015). NGRNs lacking in experience may not respond to patient deterioration with the same competence as more experienced nurses (Purling & King, 2012). NGRNs also work with uncertainty with regard to recognizing patient determination and decision‐making in complex situations (Della Ratta, 2016). As they gain experience, NGRNs become more realistic about their capabilities and more easily recognize when they require assistance from more experienced nurses.

### 2.2 Nurses' competence in relation to quality of care and patient safety

The outcome of nursing care has been shown to depend on nurses' competence, which is crucial to quality of care and patient safety (Aiken et al., 2012). Clinical competence is the ability to perform a task with a desirable outcome under various clinical context conditions in the real world. Development of clinical competence is a process all nurses undertake, from novice to experts and advances are usually stepwise rather than linear (Benner, 2001). Previous research (Burger et al., 2010) shows that NGRNs are task oriented instead of having a holistic view of patients. Missen, McKenna, Beauchamp, and Larkins (2016) showed that NGRNs lacked advanced clinical skills such as medical administration, physical assessment, emergency procedures and communication and therefore have difficulties in detecting changes in patients' conditions. Patient safety and quality of care can be jeopardized if NGRNs perform nursing care in isolation without support from experienced colleagues (Murray‐Parahi, DiGiacomo, Jackson, & Davidson, 2016).

An introduction programme can prepare and support NGRNs' transition from nursing student to the dynamic and complex role of NGRNs (Duchscher, 2009; Gardiner & Sheen, 2016; Whitehead, Owen, Henshaw, Beddingham, & Simmons, 2016). Fundamental to an introduction programme is to give support and build confidence and clinical competence in NGRNs as they develop professionally (Gardiner & Sheen, 2016; Pasila, Elo, & Kääriäinen, 2017; Walker, Costa, Foster, & de Bruin, 2017).

### 2.3 Lifelong learning and critical thinking

Lifelong learning strategies are important in nursing education programmes as well as in the professional development of Registered Nurses (RNs). Lifelong learning includes informal and non‐formal education and training, resulting in an improvement in competence (Directive 2013/55EU, 2013). Abilities such as critical thinking, reflection, questioning and enjoying learning are central to lifelong learning, which is a dynamic process in nursing that is continuous and infinite and involves gaining new perspectives on environment, knowledge, skills and interactions to develop nursing and deliver high‐quality nursing care (Davis, Taylor, & Reyes, 2014). Therefore, the ability to think critically is an important part of the development of clinical competence.

### 2.4 NGRNs' clinical competence and need for further training

Research shows that just prior to graduation, nursing students rate their clinical competence as “high” or “very high” (Gardulf et al., 2016; Theander et al. 2016), while NGRNs' self‐assessment of their competence has declined to “good” (Wangensteen, Johansson, Björkström, & Nordström, 2012). Kajander‐Unkuri et al. (2014) found that the self‐assessed competence level of NGRNs might decrease over time. A longitudinal study of NGRNs' first year working in a paediatric ward showed that their clinical competence developed most during the first 6 months (Lima et al., 2014). Previous research has focused on NGRNs' self‐assessed competence right when they are about to enter the profession (Lima, Newall, Jordan, Hamilton, & Kinney, 2016) and NGRNs with a maximum of 12 months of experience in association with variables such as ethical climate and competence (Numminen, Leino‐Kilpi, Isoaho, & Meretoja, 2015), occupational commitment, practice environment and competence (Numminen, Leino‐Kilpi, Isoaho, & Meretoja, 2016). Thus, these studies do not follow NGRNs' clinical competence over time or in the clinical context of acute care hospital settings. Lately, studies have suggested that NGRNs' nursing practice context is crucial when investigating their clinical competence (Blanchet Garneau, Lavoie, & Grondin, 2017; Kentischer et al., 2018; Lima et al., 2016). The present study is, to the best of our knowledge, the first to investigate NGRNs' clinical competence and their need for further training over time from a holistic view in various acute care hospital settings. Little is known about when and what kind of support activities and what further training will be sufficient for NGRNs to gain clinical competence when entering nursing in an acute care hospital setting.

## 3 THE STUDY

### 3.1 Aim

The aim of this study was to explore and describe changes in self‐assessed clinical competence and need for further training among newly graduated Registered Nurses during their first 15 months of work in acute care hospital settings.

### 3.2 Design

The study used a longitudinal design. The study conforms to the STROBE cross‐sectional reporting guidelines (Von Elm et al., 2014).

## 4 METHOD

### 4.1 Setting and participants

The NGRNs included in this study graduated in June 2016 from one university, and 52 of them were employed by a county council in central Sweden. In the NGRNs' first year, they participated in a mandatory clinical development programme. The county council organized the programme that included a total of 12 days of training spread over the year and consisted of: (a) the new profession; (b) clinical skills; and (c) patient safety care. The NGRNs worked in direct patient care in a central regional acute care hospital at different wards including medical, surgical, emergency, gynaecological, paediatric, psychiatric and oncological care. The NGRNs were given oral information about the study before graduating their nursing programme. At the first meeting of the clinical development programme, the NGRNs had been employed by the county council for about 2 months. They received both oral and written information about the study and were invited to participate; of the 52 NGRNs invited, a total of 45 agreed to participate in the study.

### 4.2 Data collection

Data were collected using the instrument the Professional Nurse Self‐Assessment Scale of clinical core competencies II (ProffNurse SAS II). The NGRNs answered the ProffNurse SAS II on four different occasions in connection with scheduled meetings for the clinical development programme. The baseline of the study took place at the NGRNs' first meeting of the clinical development programme, and the questionnaire was answered an additional three times after this (Table 1). Those who could not attend the data collection occasions were sent the questionnaire by post, and this was followed up by two reminders about 10 days apart. For the fourth data collection, all the questionnaires were sent out by post as the clinical development programme had finished.

**Table 1:** Overview of the four data collections, including dates, number of respondents, response rate and the length of NGRNs' work experience

Data collections	Date	*N*	Response rate	Work experience
I	2016 September	45	86%	2 months
II	2016 November	36	69%	5 months
III	2017 April	35	67%	9 months
IV	2017 October	36	69%	15 months

#### 4.2.1 Professional Nurse Self‐Assessment Scale of clinical core competencies II

ProffNurse SAS is constructed to have the nurse–patient relationship central and aims to measure nurses' clinical competence at different educational levels from a holistic and a lifelong learning perspective (Wangensteen et al., 2018). The ProffNurse SAS II has 50 items and consists of two scales, the *A scale* for self‐assessed clinical competence and the *B scale* for self‐assessed need for further training. The items in both the A and B scales have 10 response alternatives ranging from 1 = poor to 10 = very good. In ProffNurse SAS II, 44 of the 50 items have been sorted into the following six categories: direct clinical practice, professional development, ethical decision‐making, clinical leadership, cooperation and consultation and critical thinking. The six additional items that are included in ProffNurse SAS II are “I assess patients' health needs by telephone”; “I give health promotion advice and recommendations to patients by telephone,” “I give health promotion and illness preventive recommendations in accordance with national guidelines to patients”; “I have a supportive ongoing dialogue with patients about their needs and wishes”; “I focus on relatives' needs for support and guidance”; and “I report all incidents in accordance with the actual patient safety system” (Table 2).

### 4.3 Ethics

The study followed the ethical principals in accordance with the Declaration of Helsinki (Helsinki Declaration, 2013). The participants received both oral and written information about the aim of the study. They were also informed that participation was voluntary and that they could end their participation without any explanation and written informed consent was obtained from each participant. The study was given ethical approval by the Ethical Review Board (reg. no. 2011/071 and 2011/071/2).

### 4.4 Data analysis

Data were analysed using IBM SPSS version 26.0, and the significance level was set at *p* < .05 (Pallant, 2013). Per cent, mean and standard deviation were used to present data. Cronbach's alpha was calculated to assess internal consistency (Cronbach, 1951). A paired *t* test was conducted to analyse changes in scores between the four different data collections in the six components in the A and B scales (Field, 2013). There were a few internal dropouts of missing answers for both of the items in both scales when analysing data on a group level. In the paired *t* test, data require that both of the measurement occasions and all items in the respective components to be completed, which resulted in an internal dropout of 6% in data collections 2 and 3.

### 4.5 Validity and reliability

The instrument ProffNurse has been valid in previous research. In a study by Finnbakk, Wangensteen, Skovdahl, and Fagerström (2015) of nurses working in long term and home care contexts, the A scale Cronbach's alpha ranged from .77–.94. In a study by Wangensteen et al. (2018) of specialists and master's degree post‐graduate nurses using ProffNurse SAS II, the A scale showed good reliability with Cronbach's alpha at .96. In the present study, in the four data collection occasions, Cronbach's alpha ranged from .65–.92 in the six components in the A scale and ranged from .71–.96 in the six components in the B scale.

## 5 RESULTS

### 5.1 Participants

The 45 NGRNs who participated in the study had a mean age of 25.5 years (*SD* 4.5) with a range of 21–39 years, with 42 (92.9%) women and 3 men (7.1%). The response rate for the four data collections ranged from 67%–86% and is presented in Table 1.

### 5.2 Self‐assessed clinical competence (A scale) and changes within the first 15 months of work

**Figure 1:**
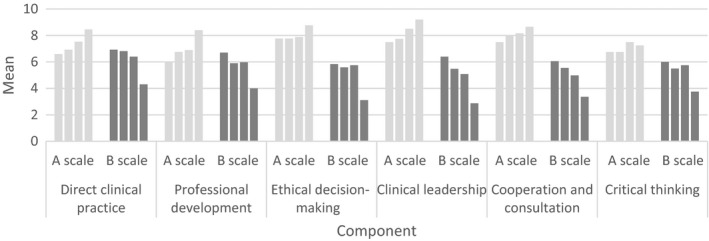
Showing the mean value for the A and B scales in the six components in ProffNurse SAS II from data collections 1–4

At data collection 1, the mean of NGRNs' self‐assessed clinical competence varied from the lowest (mean = 6.00) in the component “professional development” to the highest (mean = 7.77) in the component “ethical decision‐making.” By data collection 2, the mean score varied between 6.75 for the component “critical thinking” and 8.00 for the component “cooperation and consultation.” The mean score for all components had increased with the exception of the components “critical thinking” and “ethical decision‐making,” which had stabilized. At data collection 3, “TA” was again the component with the lowest score (mean = 6.90) and the component “clinical leadership” was scored as the highest (mean = 8.50). By data collection 4, the self‐assessed clinical competence component “critical thinking” had the lowest score (mean = 7.25) and the highest score was found in “clinical leadership” (mean = 9.20). The mean score for all components had increased between data collections 3–4, with the exception of “critical thinking” (Figure 1).

**Table 2:** Brief explanations of the six components in ProffNurseSAS II and number of items in each component

Components	Brief explanation	Number of items
Direct clinical practice	Independently identify, assess, implement and evaluate nursing in patient‐centred work. Knowledge of the effect of medicines, side effects, interactions and effects on patient health	15
Professional development	Being involved and taking responsibility for oneself, patients and the development of workplace skills	5
Ethical decision‐making	Taking ethical responsibility in the care of patients' physical, mental and social health. Taking ethical responsibility for colleagues and the work environment	10
Clinical leadership	Taking responsibility for your own decisions, actions and nursing care	4
Cooperation and consultation	Having the ability to collaborate with and consult colleagues and other staff members, delimitations to other professions	6
Critical thinking	Being able to reflect on one's own actions and evaluate work, development and visions on how nursing can be further developed for patients and the workplace	4

The highest and lowest mean score (*M*) and standard deviations (*SD*) for the six components regarding self‐assessed clinical competence for the data collections are shown in Table 3. In data collection 1, “ethical decision‐making” was the highest self‐assessed component among the NGRNs (mean = 7.77, *SD* = 0.96), and in data collection 2, the component “cooperation and consultation” was scored as highest (mean = 8.00, *SD* = 0.95). At both the data collections, 3 (mean = 8.50, *SD* = 1.10) and 4 (mean = 9.20, *SD* = 0.82) “clinical leadership” was the highest self‐assessed component in the A scale. The lowest scored self‐assessed components were “professional development” (mean = 6.00, *SD* = 1.46) at data collection 1, “critical thinking” (mean = 6.75, *SD* = 1.37) at data collection 2, “professional development” (mean = 6.90, *SD* = 1.25) at data collection 3 and “critical thinking” was the lowest scored component in data collection 4 (mean = 7.25, *SD* = 2.55) (Table 3).

**Table 3:** Presenting the NGRNs' highest and lowest scores in self‐assessed clinical competence on the A scale and in self‐assessed need for further training on the B scale for the six components

Highest scores in clinical competence A scale					Lowest scores in need for further training B scale				
Data collection	Month		*M*	*SD*	Data collection	Month		*M*	*SD*
1	2	Ethical decision‐making	7.77	0.96	1	2	Ethical decision‐making	5.85	1.88
2	5	Cooperation and consultation	8.00	0.95	2	5	Clinical leadership	5.48	1.06
3	9	Clinical leadership	8.50	1.10	3	9	Cooperation consultation	4.66	2.36
4	15	Clinical leadership	9.20	0.82	4	15	Clinical leadership	2.87	1.89

Lowest scores in clinical competence A scale					Highest scores in need for further training B scale				
Data collection	Month		*M*	*SD*	Data collection	Month		*M*	*SD*
1	2	Professional development	6.00	1.46	1	2	Direct clinical practice	6.93	1.39
2	5	Critical thinking	6.75	1.37	2	5	Direct clinical practice	6.81	1.38
3	9	Professional development	6.90	1.25	3	9	Direct clinical practice	6.40	1.44
4	15	Critical thinking	7.25	2.55	4	15	Direct clinical practice	4.30	1.49

Note

Mean value (*M*) and standard deviations (*SD*) are shown along with month the data were collected.

Changes in scores between the four different data collections in the six components are given in Table 4. There were no statistically significant changes in scores between data collections 1 and 2 in any of the components in the clinical competence A scale. Between data collections 2 and 3, there was a statistically significant increase in the components “professional development” (mean difference 0.576, *t* = 2.823, *p *< .009) and “critical thinking” (mean difference 0.607, *t* = 2.387, *p *< .024). Between data collections 3 and 4, there was a statistically significant increase in all the components of the clinical competence scores with the exception of “critical thinking,” which decreased in that period of time, see Table 4.

**Table 4:** NGRNs' self‐assessed changes between data collections for clinical competence (A scale) and need for further training (B scale) in the four data collection occasions

Component	Scale	Data collections 1–2				Data collections 2–3				Data collections 3–4			
		Mean difference	*t*‐Value	*N*	*p*‐Value	Mean difference	*t*‐Value	*N*	*p*‐Value	Mean difference	*t*‐Value	*N*	*p*‐Value
Direct clinical practice	A scale	0.338	1.768	32	.087	0.447	1.997	25	.057	0.830	4.509	30	<.001*
	B scale	−0.159	−0.643	29	.525	−0.016	−0.051	22	.960	−1.902	−2.554	28	<.001*
Professional development	A scale	0.258	0.944	31	.353	0.576	2.823	25	.009*	1.446	2.823	30	<.001*
	B scale	−0.262	−0.617	30	.542	0.539	1.719	22	.100	−1.275	−2.300	24	.031*
Ethical decision‐making	A scale	0.026	0.161	33	.873	0.086	0.621	26	.540	0.977	5.576	29	<.001*
	B scale	−0.013	−0.38	24	.970	0.273	0.758	24	.456	−2.255	−5.291	27	<.001*
Clinical leadership	A scale	0.235	1.037	34	.307	0.375	1.626	27	.115	0.867	3.839	31	.001*
	B scale	−0.969	−2.940	33	.006*	−0.267	−0.686	28	.499	−1.431	−2.659	29	.013*
Cooperation and consultation	A scale	0.343	1.606	34	.118	0.045	0.226	29	.823	0.544	2.662	30	.013*
	B scale	−0.308	−0.825	34	.416	−0.391	−0.925	26	.364	−1.577	−3.547	30	.001*
Critical thinking	A scale	0.250	1.162	33	.254	0.607	2.387	28	.024*	−0.379	−1.905	29	.067
	B scale	−0.648	−1.835	32	.760	0.046	0.116	27	.908	−0.900	−1.482	30	.149

Note

Mean score differences, *t*‐values, number of respondents and *p*‐values are shown.

* Statistically significant *p*‐values (*p* < .05).

### 5.3 Self‐assessed need for further training (B scale) and changes within the first 15 months

At data collection 1, the highest scored self‐assessed components were “direct clinical practice” (mean = 6.93) and lowest in “ethical decision‐making” (mean = 5.84). At data collection 2, the highest scored component was “direct clinical practice” (mean = 6.81) and lowest in “clinical leadership” (mean = 5.48). At data collection 3, the need for further training had increased when compared with data collection 2 in the following three components: “professional development,” “ethical decision‐making” and “critical thinking.” The highest scores were found in “direct clinical practice” (mean = 6.40) and the lowest in “cooperation and consultation” (mean = 4.66). At data collection 4, all mean scores for need for further training had decreased; however, the highest mean scores were still found in the component “direct clinical practice” (mean = 4.30) and lowest “clinical leadership” (mean = 2.87) (Figure 1).

The lowest self‐assessed mean scores in the data collections in the B scale need for further training was in the component “ethical decision‐making” (mean = 5.85, *SD* = 1.88) in data collection 1. The lowest score was “clinical leadership” (mean = 5.48, *SD* = 1.06) in data collection 2 and the lowest score “cooperation and consultation” (mean = 4.66, *SD* = 2.36) in data collection 3 and “clinical leadership” (mean = 2.87, *SD* = 1.89) in data collection 4. The top score for most needed further training was “direct clinical practice” in data collections 1–4, see Table 3.

Changes in scores between the four different data collections in the six components are shown in Table 4. In the B scale, need for further training saw a statistically significant increase between data collections 1–2 in the component “clinical leadership” (mean differences −0.969, *t* = −2.940, *p* < .006). There were no statistically significant changes between data collection two and three for any of the components. A statistically significant decrease was found between data collections 3–4 for all the components' scores of the B scale with the exception of the component “critical thinking,” which increased in that period of time (Table 4).

## 6 DISCUSSION

The aim of the study was to explore and describe changes in self‐assessed clinical competence and need for further training among NGRNs during their first 15 months of work in acute care hospital settings. All six components of clinical competence's mean scores increased over time, with a statistically significant difference between 9–15 months of experience as NGRNs, with the exception of the component “critical thinking,” which dropped during that period. The results also showed that the NGRNs' need for further training decreased over time with a statistically significant change between 9–15 months of experience as NGRNs. Numminen, Leino‐Kilpi, Isoaho, and Meretoja (2017) reported that competence development among NGRNs was modest in the beginning of their careers until the third year of working life when their competencies had been strengthened. However, they do not present data on clinical competence development during the NGRNs' first year of practice. In contrast, Lima et al. (2016) found that NGRNs working in a paediatric hospital developed most competence during the first 6 months of working as NGRNs. This indicates that clinical competence among NGRNs working in a variety of acute healthcare settings such as medical, surgical, emergency, gynaecological, paediatric, psychiatric or oncological care, takes time to develop and that the need for training is necessary during this time. These differences could be due to several reasons such as the diversity of different acute care settings and the use of different instruments to assess clinical competence. In the present study, the instrument ProffNurse SAS II was used, while the study conducted by Lima et al. (2016) used the Nurse Competence Scale (NCS). It is not easy to compare the results generated by these two instruments as they are constructed in different ways. However, there were some similarities such as critical thinking and reflection in the domain “ensuring quality” in NCS and the component “critical thinking” in ProffNurse SAS II. In the present study, the component “critical thinking” did not increase as much as the other components and the score dropped in the last data collection between 9–15 months. These results are consistent with the results presented in Lima et al. (2016) regarding the domain “ensuring quality” containing items regarding critical thinking; that domain was the lowest or second lowest self‐rated clinical competence among NGRNs working in a paediatric hospital at four data collections using the NCS scale.

### 6.1 Clinical competence

The NGRNs' self‐assessed “ethical decision‐making” was the component with the highest score at the first data collection, which is consistent with previous results that also show high scores in ethical climate with colleagues and patients (Numminen et al., 2015). The component that had the highest score after 5 months of practice as NGRNs was “cooperation and consultation”—this is the ability to collaborate with and consult colleagues. Being in a supportive atmosphere, positive socialization and being part of the team is important for NGRNs to reduce anxiety and enhance job satisfaction (van Rooyen, Jordan, ten Ham‐Baloyi, & Caka, 2018). It has, however, been reported that NGRNs are concerned that asking too many questions may be perceived as annoying and give the image that they do not have enough knowledge (Murray, Sundin, & Cope, 2019). Several studies have also noticed the importance of gaining clinical competence through support from and collaboration with experienced colleagues (Lima et al., 2016; Numminen, Ruoppa, et al., 2016; Pasila et al., 2017). On the other hand, this can be difficult to achieve as there is often a lack of experienced nurses to ask (National Board of Health & Welfare, 2015). Experienced nurses are not available to the same extent as they have been previously, and this can affect patient safety due to NGRNs having limited opportunities to ask questions. Despite the fact that there are more nurses than ever graduating from nursing programmes in Sweden (Swedish Higher Education Authority, 2018), the shortage of RNs is expected to increase further (National Board of Health and Welfare, 2015). This shortage is also used to explain NGRNs not getting any introduction (Gellerstedt, Moquist, Roos, Karin, & Craftman, 2019). The shortage along with an increased demand for nursing care among patients with complex needs is very worrying, and it is a huge challenge for healthcare systems around the world with regard to the provision of a comprehensive and safe introduction for NGRNs. Healthcare systems also need to develop sustainable ways to improve working conditions for experienced nurses to retain them in acute care hospital settings, such as offering them different career paths.

A longitudinal study conducted by Rudman, Gustavsson, Ehrenberg, Boström, and Wallin (2012) found that NGRNs' use of evidence‐based practise remained unchanged in their first 5 years of professional life. In the present study and in the study conducted by Lima et al. (2016), it was the component “critical thinking” that was not developed as much as the other clinical competences among NGRNs. The present study also found that the scores for the component “critical thinking” decreased after 9–15 months among NGRNs. This is of concern as critical thinking is an essential predictor of nurses' competence development (Rizany, Hariyati, & Handayani, 2018; Wangensteen et al., 2012) and is associated with the abilities to think intellectually, make judgements and think critically rather than length of experience (Benner, 2001). Essential elements in critical thinking and lifelong learning are the abilities to pose questions, reflect and seek learning opportunities (Davis et al., 2014). NGRNs need to be part of a supportive team where discussions with experienced colleagues and reflection on nursing care are encouraged and time is made available for them to develop critical and reflective thinking skills. In times of stress, high demands and nurse shortages, this might seem utopian, but it needs to be considered and set as a goal to strive for. This is also highlighted in the EU directive (Directive 2013/55EU, 2013), which advocates for strong teams in healthcare settings. In the light of this, teamwork and the composition of the team are becoming increasingly important. The overall responsibility for solid teams is carried by the board of the healthcare system, and head of the hospital settings and every nurse also has a responsibility to act in a professional manner. A well‐composed team of both experienced nurses and NGRNs is important for the development of clinical competence. NGRNs need to have the ability to ask questions and to feel confident that the competence needed is in the team. However, it should be noted that according to (Benner, 2001), NGRNs define themselves as novices or advanced beginners for the first 2 years of their careers and should therefore not be considered as experienced nurses for this period when composing teams. The team should give NGRNs with the opportunity to develop critical thinking skills in their daily work.

### 6.2 Need for further training

The self‐assessed need for further training was found to be lowest in “ethical decision‐making,” “cooperation and consultation” and “clinical leadership” at the four different data collection occasions. This shows that NGRNs strive to be part of their team and that they show respect and ethical values where both patients and colleagues are concerned and that they take responsibility for their own actions. These clinical competences are of great value and are the cornerstones of nursing (Swedish Society of Nuring, 2017). The component “direct clinical practice” showed the highest need for further training at all of the four data collections. This is of great concern for patient safety and quality of care as this component aims to measure NGRNs' ability to independently identify, assess, implement and evaluate nursing skills in patient‐centred work. Furthermore, knowledge of the effect of medicines, side effects, interactions and effects on patient health are included here. Clinical competences that are interconnected with direct clinical practice are essential when working in acute hospital settings with patients with complex nursing needs (Fagerström & Glasberg, 2011). The number of patients with complex nursing needs has increased due to shortened length of stay for patients in hospitals (Buchan et al., 2013; Massey et al., 2009) and in addition to this, Sweden has one of the lowest number of hospital beds per capita in Europe (OECD, 2018). The complexity in a nursing situation is affected by several factors such as instability, variability and uncertainty (Alexander & Kroposki, 2001). Instability refers to unprepared events such as sudden health deterioration. Variability is caused by several different types of health problems or several diseases and is influenced by the patient's age. Uncertainty is affected by the diversity of health problems and the patient's knowledge and ability to manage them. These factors are also influenced by interventions carried out by nurses. Nursing interventions and decision‐making in complex patient situations need to be compounded with professional judgement and experiences in addition to nursing care plans and guidelines (Alexander & Kroposki, 2001). In a recent qualitative study, NGRNs expressed that patients with multiple diagnoses were beyond their competence level and patients with sudden deterioration were challenging (Gellerstedt et al., 2019). This is not surprising as nurses with 5 years of experience have described complex patient situations as an overwhelming burden (Kentischer et al., 2018). The NGRNs included in this study have assessed that they need further training in nursing interventions in association with direct patient care. Recent studies have shown that NGRNs are afraid to make mistakes regarding medication (Halpin, Terry, & Curzio, 2017) and that the administration of medication is a cause of stress among NGRNs as they have deficient knowledge of different types of medication and their interactions (Murray et al., 2019). It is reported that 55% of NGRNs have made errors with medication leading to feelings of fear and horror as they realized they might have harmed a patient (Treiber & Jones, 2018). NGRNs experiencing a lack of patient safety and unsafe patient care due to mistakes were the most common reasons for staff turnover (Hoffart, Waddell, & Young, 2011). Higher levels of nursing competence were related to lower turnover intentions (Takase, Teraoka, & Kousuke, 2015). The results of this study show that the NGRNs need further training and support at least during their first 9–15 months of practice until their clinical competence and need for further training changes.

The NGRNs in this study were familiar with assessment tools as they had used AssCE to self‐assess their clinical competence during their nursing programme's clinical placements (Engström, Löfmark, Vae, & Mårtensson, 2017). However, Forsman et al. (2019) found that nursing students' self‐assessed clinical competence, measured using the Nurse Professional Scale, might not correspond with their results of a National Clinical Final Examination for all students as some will over or underestimate their competencies. To support the development of clinical competence among NGRNs, a self‐assessment instrument tool such as ProffNurse SAS II can be used regularly throughout the first year. However, in the light of the result from (Forsman et al., 2019), we recommend that self‐assessment of the NGRNs needs to be discussed and reflected on together with a mentor or manager to ensure self‐awareness.

### 6.3 Limitations

The instrument used in this study ProffNurse SAS II needs to be further tested in a larger sample to ensure its psychometric properties. A limitation in this study is the sample size; its small size could affect the interpretation and the generalizability of the results. Due to the number of male participants being low, no gender analysis was conducted.

## 7 CONCLUSION

Solid teams, with a mix of experienced RNs and NGRNs, are needed to keep up clinical competence and to stimulate critical thinking, reflection and lifelong learning on a daily basis. Introduction programmes with a generic design and ProffNurse SAS II can be used as assessment tools and to give direct individualized support and training to NGRNs. An assessment tool is also of value to follow up NGRNs' development of clinical competence over time as they have different needs for further training during different periods and therefore their introduction to working independently as professional nurses should be tailored to their individual needs.

## CONFLICT OF INTEREST

The authors have no conflict of interest to declare.

## AUTHOR CONTRIBUTIONS

All authors have made substantial contribution to all of the following: the conception and design of the study, data, analysis and interpretation of data, drafting the article or revising it critically for important intellectual content, final approval of the version to be submitted.
